# Study of Coronary Atherosclerosis Using Blood Residence Time

**DOI:** 10.3389/fphys.2021.625420

**Published:** 2021-05-03

**Authors:** Javad Hashemi, Bhavesh Patel, Yiannis S. Chatzizisis, Ghassan S. Kassab

**Affiliations:** ^1^California Medical Innovation Institute, San Diego, CA, United States; ^2^Cardiovascular Division, University of Nebraska Medical Center, Omaha, NE, United States

**Keywords:** residence time, wall shear stress, oscillatory shear index, coronary artery stenosis, stented bifurcation, coronary atherosclerosis

## Abstract

Computational fluid dynamic-based modeling is commonly used in stenosed and stented coronary artery to characterize blood flow and identify hemodynamics factors that could lead to coronary stenosis. One such factor is the residence time (RT), which is important for investigating stenosis and restenosis progression. The current method to calculate RT, known as the relative residence time (RRT) method, does not provide the original scale of RT and only provides a relative value. We recently introduced a novel method, designated as RT method, based on developing the advection-diffusion equation with a scalar to calculate the absolute residence time. The goal of this study was to compare both methods. Our results show that both could detect regions with a high risk of stenosis and restenosis, but the RT method is also able to show the recirculation zone using pathlines in the lumen and quantify actual RT. Moreover, RT method also provided blood flow pathlines, and is correlated to wall shear stress (WSS), oscillatory shear index (OSI), RRT, and Localized Normalized Helicity (LNH) which are other critical factors to gauge stenosis severity and assess stenting in bifurcations coronary.

## Introduction

Coronary artery disease (CAD) is the leading cause of death in US with over 840,768 deaths annually (Benjamin et al., 2019). Interventional cardiologists encounter coronary stenosis in 40–90% of such patients and have to make a decision about performing percutaneous coronary intervention (PCI, placement of a stent) (Mozaffarian et al., 2016). Stent placement is performed in 70–90% of the 1.3 million PCIs in the US (Antoniadis et al., 2015). Stenting coronary bifurcations is about 20% of all cases where a stent is implemented. About 15–20% of stented bifurcation coronaries fail between 6 months and 1 year post-intervention (Genuardi et al., 2020).

The clinical gold standard for quantification of coronary lesions is invasive measurement of Fractional Flow Reserve (FFR) (Gould et al., 1990; De Bruyne et al., 2000). FFR is measured by inserting a pressure measurement wire across a stenotic coronary lesion (Gould et al., 1990). The downstream pressure is then expressed as a fraction of the upstream pressure to get the FFR (De Bruyne et al., 2000). FFR is restricted in many clinical applications due to expensive and time-consuming procedure. Couples computational-based methods have been developed for estimating the FFR in three-dimensional (3D) reconstructed coronary based on computed tomographic coronary angiography and quantitative coronary angiography (Tu et al., 2015; Bit et al., 2020; Eswari et al., 2020). Diagnosis of coronary artery stenosis is a popular application of image-based computational fluid dynamics (CFD) and several associated indexes have been reported using CFD for quantifying coronary artery stenosis (Min et al., 2012; Papafaklis et al., 2014).

Atherosclerotic plaque development depends on different factors like blood flow, blood residence time, blood density, cholesterol concentration, and arterial geometry (Li et al., 2016). Atherosclerosis progresses based on the reaction between molecules in blood and lumen surface of artery. Atherosclerotic plaque development is complicated case study on humans and animals which takes couple of months for each case study. The principle factor for plaque creation is time reaction between molecules and surface. Therefore, the residence time of blood through coronary can demonstrate zones with high risk of atherosclerosis and help interventional cardiologist to make appropriate treatment decision for each patient.

CFD is used to quantify other hemodynamic parameters used in cardiovascular sciences such as wall shear stress (WSS), oscillatory shear index (OSI), relative residence time (RRT), and residence time (RT). RRT was introduced by Himburg et al. (2004) to study the effect of the RT of plasma and particles of the blood on the atherosclerotic process. Notably, RRT is calculated based on time averaged WSS and OSI. Most researches are focused on computational and experimental investigations of blood flow to analyze hemodynamics at the near wall region by calculating WSS (Huo et al., 2012; Koskinas et al., 2012; Antoniadis et al., 2015; Genuardi et al., 2020). WSS calculation can provide an estimation of blood flow velocity and quantify blood fractional force on wall vessel. RRT is defined as the inverse of time average WSS vector magnitude which is a qualitative factor and cannot be expressed as a scale of time (Himburg et al., 2004). The near wall fluid velocity can be quantified by the WSS vector and can measure RRT by Eulerian model (Himburg et al., 2004). Dong et al. (2013) presented a method to detect diseased carotid bifurcation by analyzing time averaged WSS, OSI, and RRT, to assess atherosclerosis. Malota et al. (2018) studied some hemodynamics indices such as the OSI, RT index, and pressure drop coefficient that are induced by change of blood flow rate, heart rate and vessel geometry which may be reflective of coronary stenosis. Shtilman et al. (1985) introduced the parameter called Localized Normalized Helicity (LNH) and Morbiducci et al. (2007) proved LNH is a useful parameter for the visualization of complex flow patterns in cardiovascular flows. The LNH is a non-dimensional factor and the cosine of the angle between the velocity and vorticity vectors and calculated using Eulerian model. Hashemi et al. (2020) assessed coronary artery stenosis by calculating Blood_RT_ based on RT. In that study, relation between WSS and blood residence time was showed, and residence time correlated with FFR significantly (Hashemi et al., 2020), but the method was not compared with RRT method.

In this study, we characterized hemodynamics changes in three patients with non-significant stenosis, significant stenosis, and bifurcation with and without stent, respectively. The goal of this work was to compare hemodynamic changes to investigate stenosis and restenosis progress in coronary arteries segment with focus on RT. Moreover, the relation between RT as new factor and other factors like RRT, LNH and OSI was studied in these three patients.

## Materials and Methods

### 3D Rendering and Patients’ Arteries

We used in-vivo methodologies (Toutouzas et al., 2015) that enabled 3D reconstruction of coronary artery bifurcation based on invasive angiography, intravascular ultrasound (IVUS), OCT. CAAS QCA-3D system (Pie Medical Imaging, Maastricht, The Netherlands) (Papafaklis et al., 2014) was used in our analysis. In this study, we used 2 left anterior descending (LAD) coronary arteries with FFR 0.96 (Patient A) and 0.66 (Patient B), and one LAD/D1 coronary artery bifurcation with and without stent (Patient C).

### CFD Modeling

**FIGURE 1:**
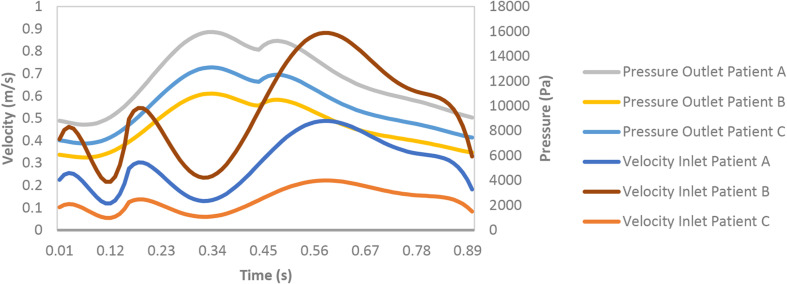
Hyperemic boundary conditions: inlet velocity and outlet pressure.

Hemodynamics of the coronary segment was simulated based on CFD using a Newtonian Single-phase 3D model. Momentum and mean residence time of each phase were calculated. The single phase 3-D Eulerian equation using laminar viscous model was solved with ANSYS Fluent 17.0. The blood viscosity defines both Newtonian (Arzani, 2018) and non-Newtonian model (Bit and Chattopadhyay, 2014), in this study; we used Newtonian model. A mixture density for blood of 1,045 kg.m^–3^ was used (Jung et al., 2006). The transient inlet phase velocity (Figure 1) waveforms for the heart coronary blood cycle in hyperemic condition for patient A and B, in baseline for patient C were matched with the mean flow rate (Davies et al., 2006) and outlet phase pressure (Figure 1) using user-defined function (UDF) and profile files which were programmed into Fluent. Mesh ANSYS 19.0 was used to generate unstructured tetrahedral cells mesh for the geometries. The optimal node count, found using sensitivity analysis, was 524,546, 1,033,714 nodes, and 252,893, for patient A, B, and C, respectively. Specifically, mesh size was successively divided by two starting from the default value suggested by ICEM software until difference in RT value between two successive meshes was <5%.

### Localized Normalized Helicity

The quantity LNH is defined as:

(1) $$L\,N\,H=\frac{\left(\nabla \times v\right).v}{\left|\left(\nabla \times v\right)\right|\,\left|v\right|}-1\leq L\,N\,H\leq 1$$

where v is the velocity vector and (∇ × v) is the vorticity. Full helical flow pattern has one LNH and symmetrical flow pattern has zero LNH.

### Residence Time Method

The RT method was introduced in our previous work (Hashemi et al., 2020). Briefly, residence time is calculated by solving an advection-diffusion equation:

(2) $$\frac{\partial C}{\partial t}+\nabla .\left(u\,C\right)=\nabla .\left(D\,\nabla C\right)$$

where C refers the concentration, D designates the diffusion coefficient, t indicates the time, and u refers to the velocity. A scalar blood residence time is defined as:

(3) $$a\,\left(x\right)=\frac{\int_{0}^{\infty }t\,C\,\left(x,t\right)\,dt}{\int_{0}^{\infty }C\,\left(x,t\right)\,dt}$$

Developing this equation with the scalar and the recognition leads to the relation:

(4) u⁢∇⁡a=∇.D⁢∇⁡a+1

This final equation provides the transport for blood residence time (Hashemi et al., 2020).

### Relative Residence Time Method

In the RRT method, the OSI and RRT are obtained from the following equations:

(5) $$\left|W\,S\,S\right|=\sqrt{{\left(W\,S\,S_{x}\right)}^{2}+{\left(W\,S\,S_{y}\right)}^{2}+{\left(W\,S\,S_{z}\right)}^{2}}$$

(6) $$O\,S\,I=\frac{1}{2}\,\left(1-\frac{\left|W\,S\,S\right|}{W\,S\,S}\right)$$

(7) $$R\,R\,T=\frac{1}{\left(1-2\times O\,S\,I\right)\times W\,S\,S}$$

Previously, RRT was calculated for a cardiac cycle period but we calculated RRT for each time step because blood residence time in the RT method is calculated for each time step and we can thus compare both in same time step (Gay and Zhang, 2009).

## Results

### Velocity

**FIGURE 2:**
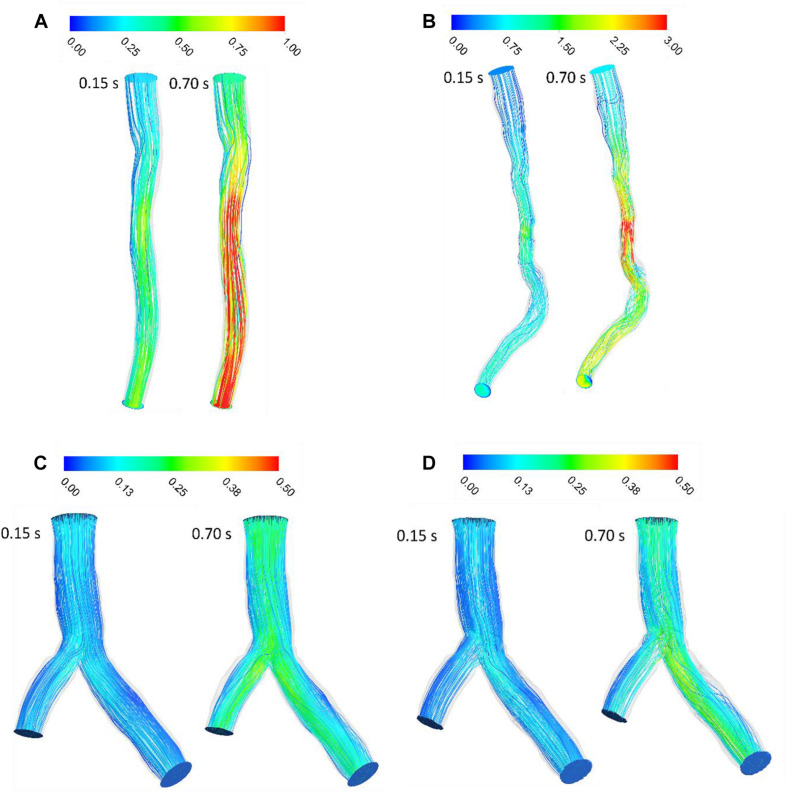
Blood velocity pathlines (m/s) **(A)** for patient A and **(B)** for patient B **(C)** for patient C without stent and **(D)** with stent during both systole (0.15 s of pulse) and diastole (0.7 s of pulse).

The velocity profile in upstream had a plug shape and a laminar character that was not fully developed for Patient A and the shape of velocity profile was plug until outlet and did not show any separation and vortex (Figure 2A). For patient B, velocity profile changed in downstream of stenosis and the pathlines show circulation after stenosis especially during diastole (Figure 2B). The pathlines for patient C showed some small vortex after bifurcation near wall during diastole; also, some circulation appeared after passing flow through the stent strut in the daughter branch during diastole while patient C without stent’s pathlines do not show the circulations (Figure 2). Figure 2 shows that increasing velocity causes circulation and separation flow in downstream of stenosis and bifurcation with stent.

### WSS

**FIGURE 3:**
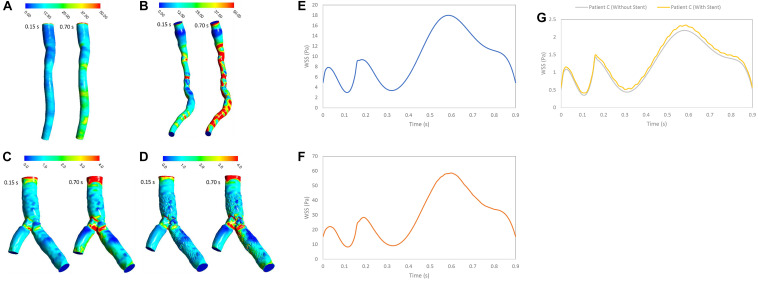
WSS (Pa) **(A)** for patient A, **(B)** for patient B, **(C)** for patient C without stent, and **(D)** with stent during both systole (0.15 s of pulse) and diastole (0.7 s of pulse), and WSS (Pa) **(E)** for patient A, **(F)** for patient B, and **(G)** for patient C in a cardiac cycle.

In stenosed and stented coronary arteries, WSS increased in diastolic phase because of increasing flow rate. WSS increases abruptly at the throat of stenosis and separated point at bifurcation. Subsequently, WSS decreased substantially because of separation flow and recirculation. The WSS contours showed that patient A did not have significant change of WSS in long direction specially in systolic phase but for Patient B, WSS increased in throat of stenosis and decreases in downstream of stenosis (Figure 3). Range of changing WSS increased with flow rate where variation of WSS in during diastole is more than systole. For patient C, bifurcation region was high WSS zones and WSS decreased along the inner strut wall, as shown in Figure 3. Moreover, stent causes increasing WSS on bifurcation segment generally (Figure 3G).

### OSI

**FIGURE 4:**
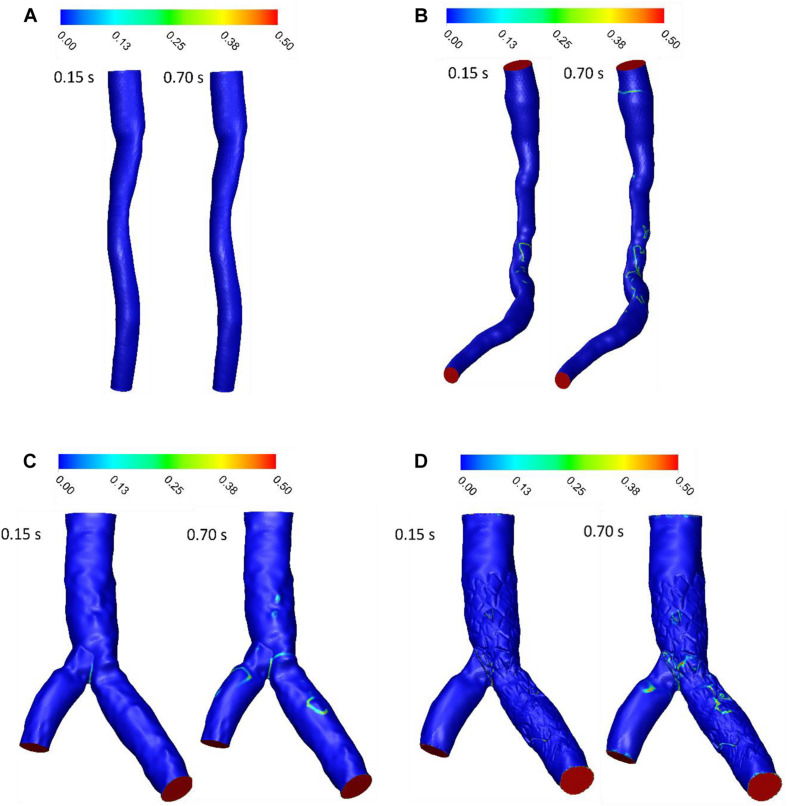
OSI **(A)** for patient A, **(B)** for patient B, **(C)** for patient C without stent, and **(D)** with stent during both systole (0.15 s of pulse) and diastole (0.7 s of pulse).

The OSI represents the oscillatory behavior of shear stress by negative and positive values. A low OSI was observed around stenosis or separated point at bifurcation. OSI increased in downstream of stenosis and branch walls in bifurcation where low WSS appeared (Figure 4). The OSI contours showed that patient A did not have any zone with high OSI, but Patient B had some zone with high OSI especially after throat of stenosis where flow was recirculated. For patient C, high OSI appeared in distal of bifurcation and first section of daughter branch where low WSS was observed. After stent replacement in bifurcation segment, OSI zones expanded where daughter branch is separated. Generally, zones with high OSI enhanced with increasing velocity and OSI in diastolic phase was higher than systolic phase.

### LNH

**FIGURE 5:**
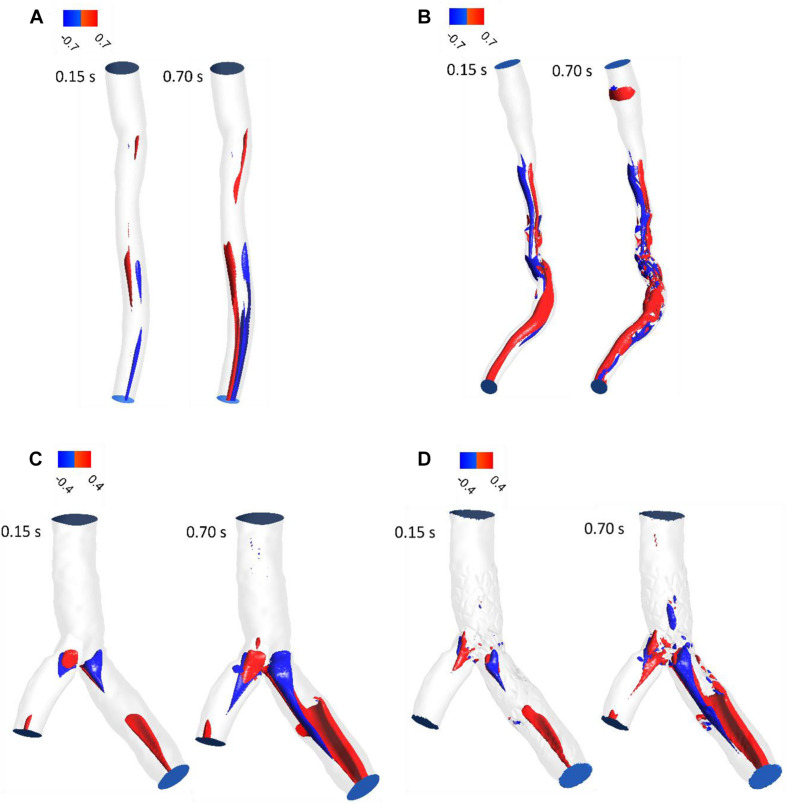
LNH isosurfaces **(A)** for patient A and **(B)** for patient B **(C)** for patient C without stent and **(D)** with stent during both systole (0.15 s of pulse) and diastole (0.7 s of pulse).

LNH is used to visualize the orientation of velocity and vorticity vectors. Figure 5 shows LNH isosurfaces by adopting a threshold value of LNH (0.7 for patient A and B, and 0.4 for patient C). LNH isosurfaces of patient A shows that helical patterns of blood flow are less marked (Figure 5A) while LNH isosurfaces of patient B appear downstream of stenosis noticeably (Figure 5B), because the flow in downstream for patient A is more separated than patient B. for patient C, large LNH isosurfaces in downstream from separation branches quantify tortuous path of the blood flow. After sent placement, the uniformity of LNH flow reduced and LNH regions in threshold increased to compare without stent segment (Figures 5C,D). Moreover, LNH isosurfaces could detect which stent struts could affect flow pattern and cause flow tortuosity. Generally, increasing velocity in diastolic phase enhanced regions with high LNH.

### Pathlines With RT Method

**FIGURE 6:**
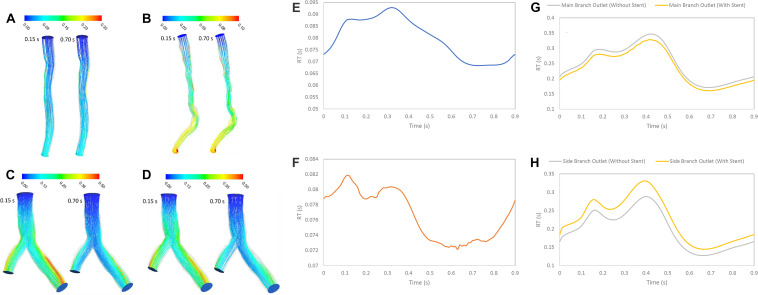
RT pathlines (s) **(A)** for patient A, **(B)** for patient B, **(C)** for patient C without stent, and **(D)** with stent during both systole (0.15 s of pulse) and diastole (0.7 s of pulse) **(E)** RT (s) on outlet for patient A, **(F)** RT (s) on outlet for patient B, **(G)** RT (s) on main branch outlet for patient C, and **(H)** RT (s) on side branch outlet for patient C in a cardiac cycle.

RT was examined for the three patients with stenosis or stent. RT calculated by scalar advection-diffusion was allowed the study of flow patterns, circulation and separation zones where it is important for stenosis progression. For patient A, the pathlines showed that the flow was not recirculated or separated, and the stream jet did not appear into the lumen (Figure 6A). For patient B, recirculation was not significant in systolic phase but in diastolic phase, flow was recirculated after throat of stenosis (Figure 6B). For patient C, the flow after separation branches had a tendency to increase along the inner walls of bifurcation and flow around outer walls of bifurcation was low. So, in diastole during, more tortuosity was observed around stent struts to compare the segment without stent where daughter branch is separated (Figures 6C,D). The average of RT for Patient A and B in a cardiac cycle is 0.0799 and 0.0767 s, respectively (Figures 6E,F). Also, the average of RT for the main branch was decreased by 6% while it was increased by 13% for side branch after stent placement in bifurcation segment (Figures 6G,H).

### RT and RRT Methods

**FIGURE 7:**
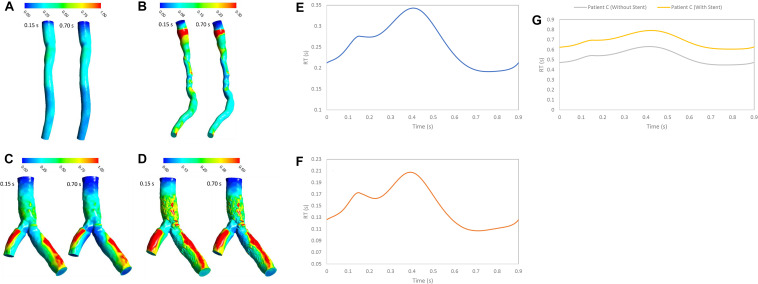
RT (s) **(A)** for patient A, **(B)** for patient B, **(C)** for patient C without stent, and **(D)** with stent during both systole (0.15 s of pulse) and diastole (0.7 s of pulse) **(E)** RT (s) on wall for patient A, **(F)** RT (s) on wall for patient B, and **(G)** RT (s) on wall for patient C in a cardiac cycle.

The RT is an important hemodynamics parameter for determining degrees of stenosis and flow pattern for investigation restenosis zone due to high reduction in WSS and low velocity in downstream of coronary artery stenosis and bifurcation region. RRT and RT contours on the wall are shown in systolic (0.15 s) and diastolic (0.7 s) phase (Figures 6, 7). RRT and RT contours appear similar with difference in order of magnitude and the results in systolic phase are more than diastolic phase generally. For patient A, the RRT did not show any change on the wall drastically and RT was observed the same (Figures 6A, 7A). RT and RRT average in a cardiac cycle is 0.2554 s and 0.190 Pa^–1^, respectively (Figures 6E, 7E). For patient B, RRT and RT showed that residence time increases in downstream of stenosis and decreases in the throat of the stenosis (Figures 6B, 7B) and RT and RRT average for cardiac cycle is 0.1486 s and 0.167 Pa^–1^, respectively (Figures 6F, 7F). As expected, the high potential restenosis zone for patient C was estimated by RRT and RT contours where RT decreases along the inner strut wall and downstream of bifurcation (Figures 6C, 7C). The average of RT and RRT for patient c with stent to compare without stent in a cardiac cycle increased 30 and 27%, respectively (Figures 6G, 7G).

## Discussion

The mechanisms of stenosis and in-stent restenosis in coronary arteries remain unclear. One of the most important phenomena to understand about stenosis and restenosis progression is hemodynamics in coronary artery. In this work, a comprehensive study of the fluid dynamics was done for understanding more about hemodynamics in different cases like non-significant stenosis, significant stenosis, bifurcation without stent and with stent. For understanding about stenosis and restenosis progression, detecting high RT zones is important for understanding stenosis and restenosis progression. The RT can assess stenosis and restenosis progression because increasing blood RT in a region can enhance the possibility of blood particles reaction with wall vessel but calculation of RT with enough accuracy is still a dilemma. In this study, we compared a recently introduced CFD-based method, called RT method, to a more established method called RRT method (Hashemi et al., 2020) for calculating RT. RT method was also used to find zones with high potential of flow stagnation and restenosis in coronary artery with stenosis or stent. While both the method measure RT using Eulerian method and measure flow stagnation based on velocity and WSS (Rayz et al., 2010; Suh et al., 2011; Long et al., 2014; Reza and Arzani, 2019), each method has a different concept of RT. The results presented in this work demonstrate the differences between the two methods. In particular, RRT method is a relative scale of RT defined as the inverse of time average WSS vector magnitude and a measure of near-wall stagnation based on WSS (Himburg et al., 2004). On the contrary, RT method is a quantitative method based on the advection of a scalar which depends on velocity vectors (Hashemi et al., 2020).

**FIGURE 8:**
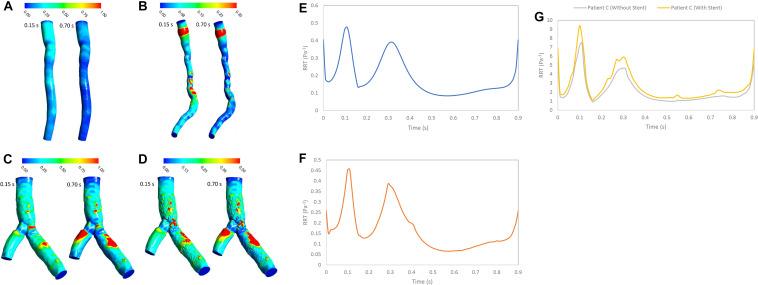
RRT (Pa^– 1^) **(A)** for patient A, **(B)** for patient B, **(C)** for patient C without stent, and **(D)** with stent during both systole (0.15 s of pulse) and diastole (0.7 s of pulse) RRT (Pa^– 1^) on the wall **(E)** for patient A, **(F)** for patient B, and **(G)** for patient C in a cardiac cycle.

Figure 3 shows that average value of WSS depends on stenosis severity, normal diameter of coronary artery, flow rate, bifurcation shape, stent structure and connection with wall (Himburg et al., 2004; Arzani et al., 2017; Chen et al., 2020). Low WSS and high OSI regions are where the atherosclerosis plaque progression may occur so, analysis of these regions is substantial for assessment of the risk of stenosis progression. But, the OSI cannot characterize flow clearly, especially when flow recirculate and separate as secondary flow. But, RRT (Figure 8) based on OSI and WSS can detect high risk of restenosis regions that have low WSS and high OSI. High OSI and RRT observed in downstream of significant stenosis and bifurcation. Malota et al. (2018) demonstrated that RRT and OSI may be useful to assess hemodynamic significance of coronary stenosis and reported the same results (Figures 4, 7) that the degree of stenosis has a significant impact on OSI and RRT where the maximum OSI and RRT appeared in an area downstream of stenosis where there also were the minimum values of WSS.

RT method results demonstrated same regions as recirculation zone in stenosis and bifurcation downstream (Figure 5). It is noteworthy that accurate 3D flow tracking immediately next to the vessel wall is a numerically challenging task especially for Eulerian method. The RT method can likely calculate flow RT next to the vessel wall. RT method can calculate flow RT based on the local velocity field. But, RRT is a measure of wall stagnation based on WSS and fails to capture the emergent transport behavior and mixing flow. RRT and RT can both detect the regions with high risk of recirculation and restenosis. A high RT and RRT in a region with low WSS may promote restenosis and recirculation that can affect blood hemodynamics and CAD progression. Both parameters had a reasonable relation and could be used as an estimate of recirculation and high RT regions. RT method has the important advantage of being able to assess flow in lumen as well as wall. RT pathlines (Figure 5) can demonstrate flow patterns and residence time in each direction how can show recirculation and separation flow. RRT (Figure 7) can, however, only characterize flow on the wall and could not show pathlines and detect recirculation zones in lumen. Another interesting advantage of RT method is calculating RT in every directions and different cross-sections of coronary artery which can characterize flow pattern and stagnation for assessment stenosis and restenosis region. Moreover, RT method could be applied in a variety of applications for both single phase and multiphase flow. RT method can calculate residence time of blood cells and proteins as multiphase flow (Hashemi et al., 2020).

The LNH is used to visualize helical flow patterns in cardiovascular system. The isosurfaces of LNH showed that large helical flow generated through coronary stenosis (Figures 5A,B) and small helical flow generated in gap between the stent struts and wall and the small region of LNH eliminated when stent removed (Figures 5C,D), in agreement with the findings by Chiastra et al. (2013). The quantity LNH is useful to prove separation and recirculation flow where RT increased. The RT pathlines (Figure 6) showed recirculation and helical flow where LNH isosurfaces appeared; so, the results demonstrated relation between LNH and RT.

Moreover, percutaneous treatment of coronary bifurcations is still a challenge for interventional cardiologists since coronary bifurcations developed plaques (Huo et al., 2012; Genuardi et al., 2020). Stents in bifurcations show a tendency for higher rates of in-stent restenosis (Chen et al., 2020). Previous studies have emphasized the significance of fluid mechanics and WSS in particular and suggested that low WSS is a major determinant of atherosclerotic plaque progression (Antoniadis et al., 2015). Moreover, Chen et al. (2009) studied the potential mechanism of restenosis between stent strut and their results are similar to high RT regions in Figure 7 where low WSS and high OSI are associated with increased inflammation and neointimal hyperplasia. Our results show that high RT and RRT were observed in low WSS region. In addition, RT method can allow visualization of recirculation flow in bifurcation downstream with low WSS. RT calculation can predict severity of restenosis on the wall artery and help In-vitro and in-vivo studies which focus on optimizing stent placement procedure in bifurcation lesion and testing new design coronary stents.

## Conclusion

We studied different cases which have potential for occurring stenosis and restenosis. We compared two methods for calculation of residence time: RT method as new method which calculated RT directly based on velocity flow and RRT method which calculated RT indirectly based on WSS and OSI. The results showed that RT and RRT methods are relevant and could both determine region with high residence time and detect the regions on the wall. RT method is a new method that was able to replicate previous hemodynamics results in coronary stenosis and in-stent restenosis. In addition, RT method allows visualization of flow pattern in lumen that is beneficial for diagnosing CAD in early stage before starting stenosis and restenosis progression. In the future, RT method could be applied for calculating RT for blood cells and proteins.

## Data Availability Statement

The raw data supporting the conclusions of this article will be made available by the authors, without undue reservation.

## Ethics Statement

The studies involving human participants were reviewed and approved by the Institutional Review Board at the University of Nebraska Medical Center approved the study; IRB number 15-159-2. The patients/participants provided their written informed consent to participate in this study.

## Author Contributions

JH contributed to conception and design of the study, acquisition, analysis, interpretation of data, and drafing and revising the manuscript. BP and YC contributed to analysis and interpretation of data and drafing the manuscript. GK contributed to concept and design of the study, interpretation of data, and revising the manuscript. All authors have participated sufficiently in the work.

## Conflict of Interest

The authors declare that the research was conducted in the absence of any commercial or financial relationships that could be construed as a potential conflict of interest.
